# Entscheidungskriterien und Patientencharakteristika zur patientenorientierten Therapie der Feldkanzerisierung: Ein standardisierter Bewertungsalgorithmus für individuelle Behandlungskonzepte

**DOI:** 10.1007/s00105-020-04731-y

**Published:** 2020-12-02

**Authors:** W. G. Philipp-Dormston, R. Aschoff, T. von Braunmühl, T. Eigentler, T. Haalck, K.-M. Thoms

**Affiliations:** 1 Fakultät für Gesundheit, Universität Witten/Herdecke, Witten, Deutschland; 2 Hautzentrum Köln, Klinik Links vom Rhein, Schillingsrotter Str. 39–41, 50996 Köln, Deutschland; 3 Klinik und Poliklinik für Dermatologie, Universitätsklinikum Carl Gustav Carus Dresden, Dresden, Deutschland; 4 Praxis für Dermatologie und Allergologie im Isarklinikum München, München, Deutschland; 5 Zentrum für Dermatologische Onkologie, Universitätsklinikum Tübingen, Tübingen, Deutschland; 6 Fachbereich Dermatologie, Ambulanzzentrum des UKE GmbH – Medizinisches Versorgungszentrum (MVZ) des Universitätsklinikums Hamburg-Eppendorf (UKE), Hamburg, Deutschland; 7 Hautkrebszentrum der UMG/Klinik für Dermatologie, Venerologie und Allergologie, Universitätsmedizin Göttingen (UMG), Göttingen, Deutschland

**Keywords:** Aktinische Keratosen, Patientenprofile, Lebensqualität, Patiententypologie, Therapieoptionen, Actinic keratosis, Patient profiles, Quality of life, Patient typology, Treatment options

## Abstract

**Hintergrund:**

Aktinische Keratosen (AK) zeichnen sich durch einen chronischen Verlauf aus, und häufig ist ein ganzes Hautareal betroffen (Feldkanzerisierung). Die patientenindividuelle Abwägung therapiespezifischer Vor- und Nachteile einer feldgerichteten Therapie ist herausfordernd.

**Fragestellung:**

Ziel der vorliegenden Arbeit war die Entwicklung und Evaluierung patientenorientierter Entscheidungskriterien, die sich für die pragmatische Einordnung einer AK-Feldtherapie im Behandlungsalltag bei Patienten mit besonderer Prädisposition zur Feldkanzerisierung eignen (Patiententyp 1 bis 3).

**Material und Methoden:**

Die Entwicklung der Entscheidungskriterien und der Patiententypologie erfolgte im Rahmen eines nominalen bzw. strukturierten Multi-level-Gruppenprozesses. Anhand der patientenrelevanten Entscheidungskriterien, der verfügbaren Evidenz aus klinischen Studien und entlang der Patiententypologie wurde ein Bewertungsalgorithmus etabliert, und feldgerichtete AK-Therapieoptionen wurden systematisch evaluiert.

**Ergebnisse:**

Als patientenrelevante Kriterien für die Therapieentscheidung wurden u. a. Effektivität, Sicherheit, Praktikabilität der Therapie, Adhärenz, Kosmetik, Patientenpräferenz und Komorbiditäten identifiziert und näher spezifiziert. In Bezug auf diese Entscheidungskriterien und Patiententypen, bei denen eine Feldtherapie vorrangig indiziert ist, erfüllte die photodynamische Therapie mit Tageslicht das therapiebezogene Anforderungsprofil in besonderem Maße.

**Schlussfolgerung:**

Die Definition von patientenrelevanten und therapiebezogenen Entscheidungskriterien in der AK-Feldtherapie erlaubt eine strukturierte und gleichzeitig praxisorientierte Herangehensweise, um spezifische Therapieoptionen einzuordnen und individuelle Therapieentscheidungen herzuleiten.

## Abstract (en)

**Background:**

Actinic keratosis (AK), which frequently affects larger skin areas (field cancerization), is characterized by chronic course. Weighing therapy-specific advantages and disadvantages of field-oriented therapy for individual patients is challenging.

**Objectives:**

The main objective was the development of patient-oriented decision criteria for the pragmatic classification of field-directed AK treatment options in patients with a predisposition for field cancerization (patient types 1–3).

**Materials and methods:**

The development of the decision criteria and patient typology was based on a nominal respectively structured multilevel group process. The developed algorithm was then subsequently applied for the systematic evaluation of field-directed AK therapies.

**Results:**

Patient-relevant criteria for the treatment decision included (among others): effectiveness, selectivity, safety, duration of therapy, cosmesis, patient preference and comorbidities. With regard to the decision criteria and patient types in which field therapy was the treatment of choice, daylight photodynamic therapy notably met the requirement profile.

**Conclusion:**

The definition of patient-relevant and therapy-related decision criteria in AK field therapy allows a systematic yet practice-oriented approach to classify specific treatment options and to derive individual treatment decisions.

Aktinische Keratosen treten häufig chronisch rezidivierend und multilokulär im Sinne einer Feldkanzerisierung auf. Viele der Überlegungen, die wir im Alltag zur Einschätzung des Therapieerfolgs bzw. einer Therapieentscheidung heranziehen, wurden bislang nicht systematisch evaluiert. Um eine gleichermaßen patientenbezogene wie sinnvolle und nachhaltige Therapieentscheidung treffen zu können, bietet sich die Erarbeitung von Patiententypologien und patientenorientierten Entscheidungskriterien an.

## Hintergrund

Aktinische Keratosen (AK) gehören zu den häufigsten Hautläsionen im dermatologischen Alltag [1]. In einer Studie aus Deutschland betrug die standardisierte Prävalenz der AK 2,7 % auf Basis einer großen Kohorte von Arbeitnehmern bzw. 1,8 % auf Basis von 4,5 Mio. Versicherungsdaten. Die Häufigkeit stieg mit zunehmendem Alter an – von 1,6 % im Alter von bis zu 60 Jahren auf 8,2 % bei den 80- bis 89-Jährigen [2].

AK entstehen in erster Linie durch die kumulative Exposition gegenüber ultravioletter (UV) Strahlung und finden sich daher überwiegend an chronisch lichtexponierten Hautarealen, wie z. B. Stirn, Halsbereich, Arme oder Handrücken [3, 4]. Faktoren wie steigende Lebenserwartung, geändertes Freizeitverhalten, Umweltveränderungen und die damit verbundene wachsende kumulative Sonnenlichtexposition dürften zu einer weiterhin wachsenden Prävalenz der AK beitragen [5, 6]. Eine hohe Prävalenz von AK lässt sich zudem bei Patienten beobachten, die auf eine chronische Immunsuppression angewiesen sind [7].

AK-Läsionen können in unterschiedlichen Stadien (kategorisiert z. B. klinisch I–III nach Olsen et al. [8] oder histologisch nach Röwert-Huber et al. [9]) auftreten, wobei sie häufig ein ganzes Hautareal (Feldkanzerisierung) betreffen, das neben sichtbaren auch subklinische AK-Läsionen umfasst, bei denen die gleichen molekulargenetischen Veränderungen vorliegen [5, 10]. Auch wenn es derzeit nicht möglich ist, das individuelle Progressionsrisiko einer spezifischen AK-Läsion in ein Plattenepithelkarzinom (SCC) vorherzusagen, müssen AK als wichtiger prädiktiver Marker für die Entwicklung eines nichtmelanozytären malignen Hauttumors (NMSC) bzw. als häufigste Vorstufe für ein SCC gewertet werden [11, 12]. Als Behandlungsoptionen stehen chirurgische, physikalisch destruierende Maßnahmen oder topische arzneimittelbasierte Verfahren zur Verfügung. Zur Entwicklung eines allgemeingültigen Therapiestandards haben verschiedene Arbeitsgruppen eigene Leitlinien entwickelt [3, 6, 13]. Die aktuelle deutsche S3-Leitlinie „Aktinische Keratose und Plattenepithelkarzinom der Haut“ [14] wurde im März 2020 (Leitlinienreport; Juni 2019) erstellt und hat eine angedachte Gültigkeit von 5 Jahren. Ein von Onkoderm e. V. entwickelter Therapiealgorithmus zur Behandlung von aktinischen Keratosen und insbesondere der Feldkanzerisierung zeichnet sich durch eine besonders hohe Praxisrelevanz aus [15].

Angesichts der Vielfalt der verschiedenen Therapieoptionen einerseits und der Heterogenität des Patientenspektrums andererseits setzt die Therapieplanung die Kenntnis therapiespezifischer Vor- und Nachteile und ihre individuelle Abwägung voraus. Aspekte wie Lebensqualität, Praktikabilität der Therapie, aber auch Therapieadhärenz, kosmetisches Ergebnis, Patientenpräferenz, Komorbiditäten oder Selektivität einer Therapie sind neben der Effektivität und Sicherheit weitere wichtige Faktoren. Ziel dieser Arbeit war es daher, Kriterien zu erarbeiten, die sich für eine pragmatische Einordnung der zur Verfügung stehenden AK-Feldtherapien eignen, und diese anhand dreier definierter AK-Patientenprofile zu evaluieren, um damit patientenorientierte Therapieentscheidungen treffen zu können.

## Methodik

Die Arbeitsgruppe setzte sich aus Experten auf dem Gebiet der AK-Therapie zusammen (*n* = 6), welche die ambulante Versorgung von NMSC-Patienten an allen Versorgungsstrukturen (Universitätsklinik, medizinisches Versorgungszentrum, niedergelassene Facharztpraxis) länderübergreifend (Dresden, Hamburg, Göttingen, Köln, München, Tübingen) abbilden. Alle Teilnehmer des Expertenpanels haben einen dermatoonkologischen Schwerpunkt und verfügen über langjährige und regelmäßige Anwendererfahrungen in der Therapie der Feldkanzerisierung. Die Mitglieder der Arbeitsgruppe wurden gebeten, patientenrelevante Aspekte in der aktuellen Therapielandschaft der AK zu analysieren und auf Basis von erstellten Fallvignetten zu folgenden Fragen Stellung zu beziehen:

1. Nach welchen patientenrelevanten Kriterien sollte die Therapiewahl beim AK-Patienten erfolgen? (Was ist aus Patientensicht wichtig? Was hilft im Therapiealltag?)
2. Welche Patienten sind im Therapiealltag häufig anzutreffen, und bei welchen Patienten ist eine feldgerichtete Therapie sinnvoll? (Welche Patientenmuster weisen ein erhöhtes Risikoprofil für die Feldkanzerisierung auf? Welche AK-Patientenrisikoprofile stellen eine therapeutische Herausforderung dar?)
3. Welches Therapie- und Einsatzprofil ergibt sich aus der beispielhaften Beurteilung einer AK-Therapieoption auf Basis der oben definierten Entscheidungskriterien und Patiententypologien? Als praxisrelevante Beispiele dienten die photodynamische Therapie (PDT) sowie Behandlungen mit Imiquimod, Ingenolmebutat und Diclofenac-Natrium. (Erfüllt die jeweilige Therapie das therapeutische Anforderungsprofil für diese Patienten?)

Die Fragestellungen 1 und 2 wurden beim ersten Arbeitstreffen im Oktober 2017 im Rahmen eines nominalen Gruppenprozesses sowie mehrerer folgender Telefonkonferenzen formuliert. Auf Basis eines Ergebnisprotokolls und der Statements wurden Entscheidungskriterien und Patiententypen in einer ersten Entwurfsversion zusammengefasst und von den Mitgliedern der Arbeitsgruppe kommentiert. Beim zweiten Arbeitstreffen im Oktober 2018 wurde die kommentierte Version im Rahmen eines strukturierten Gruppenprozesses insbesondere hinsichtlich Fragestellung 3 bearbeitet. Ergebnisse und Kommentare wurden 2019 in einem strukturierten Multi-level-Prozess eingearbeitet, 2020 erneut evaluiert und kommentiert, bevor die endgültige Version in der vorliegenden Form konsentiert wurde. Im Rahmen der erweiterten und vertieften Evaluation wurde eine exemplarische Eignungs- und Fallbewertung zur Tageslicht-PDT (Daylight-PDT, DL-PDT) durchgeführt. Der Hersteller des Ingenolmebutat-basierten Arzneimittels hat 01/2020 in Abstimmung mit der EMA (Europäische Arzneimittel-Agentur) beschlossen, die Zulassung ruhen zu lassen und das Präparat in Deutschland nicht mehr auszuliefern, weshalb diese ebenfalls untersuchte Behandlungsoption keinen Einzug in die finale Fassung fand.

## Ergebnisse

### Entscheidungskriterien für die Therapiewahl

Die Tab. 1 führt die gemäß dem Konsens in der Arbeitsgruppe wesentlichen, therapiebezogenen Kriterien auf, die patientenrelevante Fragestellungen umfassen und eine individuelle Beurteilung einer für den Therapiealltag gut geeigneten AK-Therapieoption ermöglichen. Ist der Patient präferenziell auf eine feldgerichtete Therapie der AK angewiesen (bei multiplen AK-Läsionen/Feldkanzerisierung), sollte die Therapieoption auch subklinisch betroffene, chronisch UV-geschädigte Hautareale mit erfassen, aber gesunde Zellen weitestgehend intakt lassen (Selektivität). Rezidivpräventive Eigenschaften, Sicherheit (Fokus auf Lokalreaktionen, Schmerzhaftigkeit, systemische Toxizität v. a. bei immunsupprimierten Patienten), Wechselwirkungen, Wiederholbarkeit und Dauer der Therapie oder Planbarkeit sind auch dem chronischen Verlaufscharakter der AK geschuldet, der oftmals wiederholte Therapiezyklen erforderlich macht. Dies impliziert, dass die Behandlung auch bei wiederholter Anwendung für den Patienten sowohl im Hinblick auf unerwünschte Nebenwirkungen, kosmetisches Ergebnis oder Praktikabilität akzeptabel ist und sich nicht ungünstig auf die Lebensqualität auswirken sollte.

**Table 1:** 

Entscheidungskriterium	Definition
*Effektivität*	Evidenz zur Wirksamkeit?
*Selektivität*	Werden subklinische Läsionen mit erfasst? Bleiben gesunde Zellen weitestgehend intakt?
*Nachhaltigkeit*	Evidenz zu verzögerter oder reduzierter Rekurrenz verfügbar? Reduktion der Progressionsrate zum SCC?
*Sicherheit*	Gibt es systemische Resorption/Toxizität? Gibt es Lokalreaktionen? Ist die Behandlung schmerzhaft?
*Wechselwirkungen*	Welche Wechselwirkungen sind zu beachten?
*Wiederholbarkeit*	Wiederholte Anwendung möglich und sinnvoll?
*Therapiedauer*	Dauer der Anwendung?
*Planbarkeit*	Wie gut lassen sich die Therapiedauer und voraussichtliche Abheilung der Läsionen mit dem Lebens- und Berufsalltag vereinbaren?
*Kosmetik*	Wie ist das kosmetische Ergebnis langfristig?
*Lebensqualität*	Wie wirkt sich die Therapie auf die Lebensqualität aus?

*SCC* Plattenepithelkarzinom

### Patiententypologien in der ambulanten Versorgung

Die Teilnehmer der Arbeitsgruppe haben prägnante Patientenprofile erarbeitet und 3 typische Risikopatiententypen (Typ 1 bis 3) mit Prädisposition zur Feldkanzerisierung definiert (Tab. 2). Bei diesen Patiententypen ist eine feldgerichtete Therapie vorrangig indiziert:

**Table 2:** 

	Typ 1 Leistungsorientiert, aktiv und gut informiert	Typ 2 Outdoor-Worker	Typ 3 Aufgeklärter, immunsupprimierter Patient
*Geschlecht*	Männer und Frauen	Überwiegend Männer	Männer und Frauen
*Bildungsstand*	Hoch	Niedrig bis mittel	Alle
*Sozioökonomischer Status*	Hoch	Niedrig bis mittel	Alle
*Kumulative UV-Exposition*	Hoch (+) durch aktives Freizeitverhalten	Hoch (+) durch berufliche Tätigkeit	(+/−)^a^
*Informationsstand*	Umfassender Aufklärungsbedarf über Erkrankung und Therapiemöglichkeiten/Risiken	Tendenziell geringere Krankheits- und Therapieeinsicht	Hoher Wissensstand, Routine im Umgang mit Ärzten/Diagnosen; hohe Therapieeinsicht
*Kosmetischer Anspruch an Therapie*	Hoch	Niedrig bis mittel	Niedrig bis hoch
*Patientenwünsche/Besonderheiten*	Kurze Ausfallzeiten, um berufliche/private Einschränkungen zu minimieren	Kurze Therapiedauer (ggf. eingeschränkte Therapieadhärenz)	Gespräch auf Augenhöhe, Sicherheit in der AK-Therapie (ohne systemische Nebenwirkungen)

^a^Patienten sind dazu angehalten, im Freien auf konsequenten Sonnenschutz zu achten

- Typ 1: z. B. der 61-jährige leitende Angestellte, der „schon immer sportlich aktiv“ war, umfassend über die Erkrankung, Präventions- und Therapiemöglichkeiten aufgeklärt werden möchte und an „modernen“ Therapieoptionen interessiert ist, die sich zügig umsetzen lassen, sich kosmetisch nicht nachteilig auswirken und mit einem vollen Terminkalender zu vereinbaren sind;
- Typ 2: z. B. der 50-jährige subjektiv beschwerdefreie Dachdecker, bei dem im Rahmen des Gesundheitschecks zufällig eine AK diagnostiziert wird, der bezüglich der Therapienotwendigkeit wenig einsichtig ist und bei längerer Therapiedauer voraussichtlich non-adhärent sein wird;
- Typ 3: z. B. der 55-jährige, vor 4 Jahren nierentransplantierte Patient, der bei der regelmäßigen Check-up-Untersuchung mit AK diagnostiziert wurde, gut über das erhöhte Hautkrebsrisiko unter Langzeitimmunsuppression informiert ist und das Für und Wider der AK-Therapieoptionen abwägen möchte.

### Erfüllt die photodynamische Therapie mit Tageslicht das Anforderungsprofil einer patientengerechten Feldtherapie?

Als praktisches Anwendungsbeispiel diente die photodynamische Therapie mit Tageslicht (DL-PDT), der in dieser Form jüngste Therapieansatz zur feldgerichteten AK-Therapie. Die Bewertung der DL-PDT auf der Basis der Studienevidenz und entlang der entwickelten Kriterien für eine patientenindividuelle Therapie, wurde systematisch für die jeweiligen definierten Patientenprofile durchgeführt.

Hinsichtlich der Risikopatiententypologie für die Feldkanzerisierung erfüllte die DL-PDT die Kriterien für eine effektive Therapie mit vergleichsweise geringen Rezidivraten [16, 17] und trägt damit zu einer signifikanten Steigerung der Lebensqualität bei [18].

Ein besonderer Vorteil des photodynamischen Wirkmechanismus ist seine hohe Selektivität, welche die Behandlung großer Hautareale erlaubt, die Mitbehandlung subklinischer AK-Läsionen ermöglicht und somit einen hohen Stellenwert in der Prävention von NMSC aufweist [19]. Hiervon profitieren alle Patiententypen, wenn auch der Nutzen gerade für den aufgeklärten, immunsupprimierten Patienten (Typ 3) besonders groß ist.

**Figure 1:**
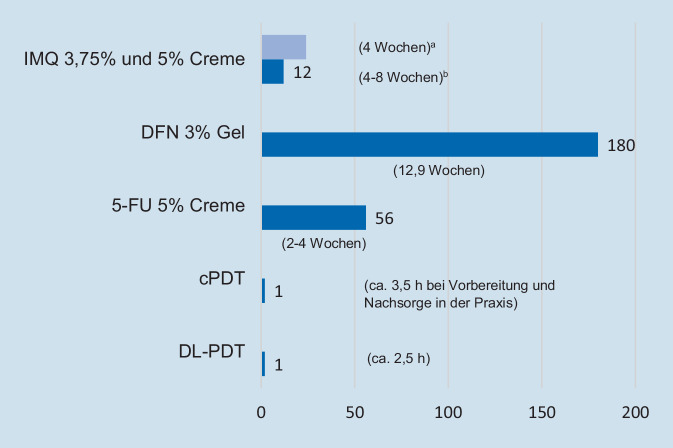

Die kurze (plausibel vermittelbare) Therapiedauer bzw. Anwenderfreundlichkeit steigert beim Typ 2 die Behandlungsadhärenz, ermöglicht dem beruflich stark eingespannten Typ 1 eine kurze Ausfallzeit und lässt sich ebenfalls in hohem Maße mit der erhöhten Arztbesuchfrequenz des Typ 3 vereinbaren. (Abb. 1 stellt den zeitlichen Aufwand der zugelassenen Therapieoptionen der Feldkanzerisierung vergleichend dar.)

Da die Belichtung im Vergleich zur konventionellen Rotlicht-PDT aufgrund der ausbleibenden Anreicherung von Protoporphyrin IX weitestgehend schmerzfrei ist, eignet sich die DL-PDT in besonderer Weise für große Hautareale, was wiederum die Notwendigkeit für Mehrfachbehandlungen reduziert [20].

Für die Sicherheit spricht, dass wegen fehlender systemischer Resorption diesbezüglich keine Risiken zu erwarten sind [16, 17], wovon insbesondere der Typ 3 profitiert, wohingegen das fehlende Narbenrisiko und die hervorragenden kosmetischen Behandlungsergebnisse einen besonderen Mehrwert für den Typ 1 darstellen [21].

Da der Zeitpunkt und die Dauer der lokal auftretenden Hautreaktionen weitestgehend vorhersagbar sind, ist bei der DL-PDT auch die Planbarkeit der Therapie gegeben, was ebenfalls für den Typ 1 von besonderer Bedeutung ist [20].

Allerdings bedarf es vor der ersten Behandlung einer ausführlichen Aufklärung zu Wirkmechanismus und Therapieablauf. Ein entscheidender Nachteil der PDT mit natürlichem Tageslicht sind die jahreszeitlichen und klimatischen Einschränkungen (März bis Oktober, Außentemperaturen >10 °C, kein Regen). Hier hat sich die artifizielle DL-PDT, auch simulierte Indoor-DL-PDT genannt, bewährt, die neben dem Vorteil der Wetterunabhängigkeit auch keinen zusätzlichen Sonnenschutz erfordert, da die gängigen Belichtungseinheiten zwar das Tageslichtspektrum simulieren, allerdings ohne den ultravioletten Anteil [22]. Nicht zuletzt aufgrund der besseren Standardisierbarkeit, der reproduzierbaren gleichen Wellenlängen und der Lichtenergie sowie des Ausbleibens unvorhersehbarer äußerer Umwelteinflüsse ist diese Art der Tageslicht-PDT als besonders sicher und effektiv zu werten, was erstmals durch die klinischen Daten der Arbeitsgruppe von U. Reinhold mit der ersten standardisierten Indoor-Lichteinheit überhaupt beschrieben werden konnte [23]. Heutzutage stehen verschieden LED-Lichtquellen zur artifiziellen Tageslicht-PDT in Innenräumen zur Verfügung. Eine aktuelle Studie aus der Arbeitsgruppe von T. Dirschka hat den hohen Stellenwert dieser Art der Feldtherapie nochmals bestätigt [24]. Das hier angewandte Behandlungsprotokoll wurde mit 1 h Aminolävulinsäure(ALA)-Inkubation, gefolgt von einer weiteren Stunde Belichtung in einer neuen Tageslichtbehandlungskabine (20.000 lx, emittierte Wellenlängen an die PP[Protoporphyrin] IX-Absorptions-Peaks angepasst: 457 nm, 523 nm, 593 nm, 631 nm) durchgeführt. Dieses adaptierte neue Behandlungsprotokoll erwies sich als sicher, beinahe schmerzfrei und sehr effektiv im Hinblick auf die Reduktion des „AK area and severity index“ (AKASI) und der Läsionsanzahl [24].

## Diskussion

Zusammenfassend wurde mit der vorliegenden Arbeit ein patienten- und praxisorientierter Algorithmus zur Therapieentscheidung in der AK-Feldtherapie entwickelt. Die Charakterisierung von Patiententypen nach psychosozialen Faktoren kann das Patientenmanagement bei chronischen Erkrankungen erleichtern, indem sie dem Behandler Selektionskriterien aufzeigt, die über die alleinige Erhebung von biomedizinischen Parametern hinausgehen. Wie die Evaluation am Beispiel der DL-PDT zeigt, lassen sich therapie- und patientenspezifische Aspekte anhand der Entscheidungskriterien pragmatisch einordnen und bezüglich der vorliegenden Studiendaten diskutieren. Unseres Wissens ist ein vergleichbarer Entscheidungsalgorithmus bisher nicht verfügbar gewesen.

**Figure 2:**
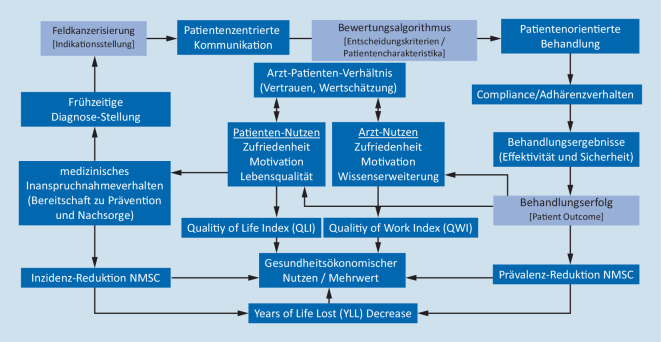

Die Charakterisierung von Patiententypen, die auch psychosoziale Merkmale (z. B. Typ-A- und Typ-D-Persönlichkeit) mit einbezieht, erfährt bei chronischen, kardiovaskulären Erkrankungen nicht nur das zunehmende Interesse der Patienten, Leistungserbringer oder Kostenträger, sondern hat bereits Eingang in Leitlinien und Positionspapiere der Fachgesellschaften gefunden. Der integrative Ansatz kann eine patientenzentrierte Kommunikation fördern, die wiederum positive Effekte auf Patientenzufriedenheit, Adhärenz- und medizinisches Inanspruchnahmeverhalten oder auf Behandlungsergebnisse haben kann [25, 26]. Mögliche Ansatzpunkte, die auch beim dermatologischen AK-Patienten für eine Stratifizierung nach Patiententypologie sprechen würden, ergeben sich aus der epidemiologischen Entwicklung, Chronizität der Erkrankung, Heterogenität des Patientenpools sowie der fehlenden Möglichkeit, zwischen AK-Läsionen mit und ohne Rezidiv- oder Progressionsrisiko zu unterscheiden. Trotz der zum Teil umfangreich publizierten Studienliteratur zu einzelnen Therapieverfahren der AK lassen sich solche Ergebnisse nur bedingt miteinander vergleichen, da u. a. verschiedene Endpunkte gewählt wurden bzw. kritische Endpunkte fehlen, die Studien nur selten direkte Vergleiche enthalten oder über keine ausreichende Power verfügen. Die Komplexität eines multimodalen patientenorientierten Ansatzes zum Management der Feldkanzerisierung und der mögliche Stellenwert eines Bewertungsalgorithmus im ganzheitlichen Kontext sind in Abb. 2 dargestellt.

Der systematisch entwickelte und konsentierte Bewertungsalgorithmus soll dabei Entscheidungskriterien aufzeigen, um anhand der existierenden Studienlage das jeweils geeignete Therapieverfahren auszuwählen und hierdurch den Behandlungserfolg bei der Feldkanzerisierung durch eine patientenorientierte Therapie zu steigern.

## Fazit für die Praxis

- Patientenorientierte Entscheidungskriterien können die Einschätzung des praktischen Stellenwerts einer Therapieoption bei AK(aktinische Keratosen)-Patienten erleichtern.
- Die Einordnung einer AK-Therapieoption entlang der Patiententypen mit erhöhter Prädisposition zur Feldkanzerisierung sowie unter Berücksichtigung der aktuellen Studienlage bietet sich für die Evaluierung verschiedener topischer Therapiemodalitäten an.
- Ähnlich wie bei verschiedenen chronisch internistischen Erkrankungen bereits zu beobachten, lassen sich Patiententypologien möglicherweise auch bei der chronischen AK zur Umsetzung integrativer Behandlungskonzepte nutzen.
